# Changes in water quality condition at sequential monitoring stations based on IRWQI along the Little Zab River in Northwestern Iran

**DOI:** 10.1038/s41598-025-14982-1

**Published:** 2025-08-12

**Authors:** Tayebeh Irani, Raoof Mostafazadeh, Saeid Mousavi Moghanjoghi

**Affiliations:** 1 Department of Range and Watershed Management, Faculty of Natural Resources, Urmia University, Urmia, Iran; 2 Department of Natural Resources and Member of Water Management Research Center, Faculty of Agriculture and Natural Resources, University of Mohaghegh Ardabili, Ardabil, 5619911367 Iran; 3 Department of Environmental Sciences, Islamic Azad University Damavand Branch, Tehran, Iran

**Keywords:** Water quality index, Monitoring stations, Water pollution, Spatio-temporal variation, Statistical analysis, Environmental chemistry, Environmental impact, Hydrology, Environmental chemistry

## Abstract

Water quality monitoring is essential for understanding ecosystem health and guiding effective water management strategies. In particular, water quality indices (WQI) are crucial tools for assessing the status of surface water bodies, providing a simplified measure of water quality across various parameters along sequential monitoring stations along a river system. This study aims to assess the spatial and temporal variations in water quality along the Little Zab River in northwestern Iran, using the Iranian Water Quality Index (IRWQI). This study examines water quality at four sequential monitoring stations along the river using the IRWQI, incorporating critical water quality parameters. Data for the analysis were collected from 2015 to 2024 and analyzed using non-parametric tests, including Kruskal–Wallis, to detect significant variations in water quality across the monitoring stations. According to the results, water quality varies across the stations. Water quality varies across stations. The upstream Mirabad-Upland station has a low IRWQI (56.51) due to wastewater from Chaku village. It improves at Grzhal-Bridge (60.04) via self-purification but declines at Nalas (57.35) due to pollution from Vavan village and agriculture. Sardasht-Dam records the highest IRWQI (64.46), likely benefiting from self-purification and cleaner inflows. Mirabad-Upland has “Fairly Good” to “Moderate” water quality. Grzhal-Bridge improves slightly, with some “Good” and “Very Good” cases, but occasional “Bad” levels. Nalas declines to mostly “Bad” and “Fairly Bad,” likely due to pollution. Sardasht-Dam shows partial recovery, though some “Bad” cases persist. Overall, water quality worsens downstream due to pollution and hydrological changes. The Little Zab River’s water quality followed a seasonal pattern, improving in wet months and declining in dry months due to flow changes and pollutant levels, indicating the need for year-round monitoring. The results suggest that localized pollution sources, such as wastewater discharge impact water quality, particularly in upstream sections. These results indicate the need for improved pollution control.

## Introduction

### Background

The preservation of river water quality plays a crucial role in the socio-economic development of communities and the survival of many ecosystems^1^. Urban and rural development, along with factors such as climate change, pose significant threats to the water quality of these environments^2,3^. Therefore, monitoring and controlling surface water to ensure its high quality for various applications is vital^4^. Regular assessment and monitoring of river water quality parameters, along with selecting key monitoring stations that effectively capture water quality variability, are essential for sustainable management^5–7^. Water quality in any location reflects the impact of various factors such as geology, climatic conditions, and human pollution sources^8,9^. Pollution and declining water quality reduce usable resources, hinder economic growth, and endanger health, stressing the need for effective monitoring and management to ensure sustainability^10,11^. Monitoring water quality often generates complex data, providing meaningful perspectives into the behavior of water resources that require appropriate methods for analysis and interpretation^12^. Classification, simulation, and statistical analysis of the data are key aspects of water quality evaluation^13^. Water quality indices are a suitable and simple tool for determining the status and conditions of water quality^14,15^. These indices, which integrate multiple water quality parameters through mathematical relationships, reflect the quality conditions in a way that can be categorized on a relative scale^16^. Water Quality Index (WQI) is an effective and efficient tool for assessing the quality of surface and groundwater^17^. Different water quality indices, by introducing cumulative functions, convert a wide range of physical, chemical, and biological parameters into a single value for classifying water body quality^18,19^. Since the 1960s, when the first model based on ten water quality parameters was proposed^20^, water quality indices have been a simple and effective tool for determining water quality status. Water quality indices have been very useful in monitoring programs to assess ecosystem health and can serve as criteria for successful evaluation and appropriate management strategies to improve water quality^21^.

### Literature review

To assess water quality variations using the IRWQI index and identify pollutant sources, extensive studies have been conducted, some of which are presented below. Shourian et al.^22^ modeled eutrophication in Ilam Reservoir, Iran, finding it to be mesotrophic to eutrophic. They showed that cutting nutrient loads by half and releasing water in fall improves water quality. Moridi and Yazdi^23^ modeled sediment concentration and flushing strategies for Dez Reservoir, Iran. They recommended flushing in March with set discharge and concentration limits to protect downstream ecosystems while preserving reservoir storage. Khalife & Khoshnazar^24^ examined the water quality of the Zarrineh River in the catchment area of Lake Urmia using the IRWQI index and found that no station fell into the “Very Bad” or “Very Good” ranges. Only in spring, station number 16 showed poor water quality and improvement in EC-related coefficients. They showed the advantage of this index over others, as it takes into account domestic wastewater parameters in its calculations. Nizar et al.^25^ used the WQI index to assess the quality of two rivers in Kelantan, Malaysia, and found that the water quality was poor. Shahsavar et al.^26^ evaluated the annual water quality of the Karde Dam using the IRWQIsc and NSFWQI indices. Their results showed that the IRWQI index for the Karde Dam in spring, summer, fall, and winter was 55.43, 49.25, 57.61, and 60.9, respectively, indicating “Fairly Good,” “Moderate,” “Fairly Good,” and “Fairly Good” water quality. According to the NSFWQI index, the water quality values for spring, summer, fall, and winter were 86.4 (Good), 81.28 (Good), 84.48 (Good), and 96.64 (Excellent). A comparison of the two indices revealed that the IRWQIsc provides a more accurate assessment of water quality, offering more precise and comprehensive judgments about the water quality at Karde Dam. Moridi^27^ proposed a bankruptcy method using Qual2K and particle swarm optimization to allocate pollution loads in a polluted river in northern Iran. The method improved conflict resolution among polluters and enhanced water quality management. Rezaeiarshad et al.^28^ evaluated water quality in the Kan River basin, analyzing surface and groundwater indices, WQI, and nitrate-related health risks. Despite infant HQ and ELCR values exceeding USEPA limits, water quality ranged from medium to good and met national standards, making it suitable for drinking. Kwon & Jo^29^ studied the water quality of the Nam River in South Korea using water quality indices and multivariate statistical analysis. They found that human activities and untreated wastewater from industries were significant contributors to the declining water quality of the river. Gabr & Soussa^30^ assessed the water quality in the southeastern Nile Delta of Egypt using water quality indices. Their results indicated that the water in the study area was classified as poor quality, with pollution levels increasing due to agricultural and industrial activities.

Seifollahi et al.^31^ aimed to map the water quality of surface waters in the Mashlak River near a waste disposal site, using the Iranian Surface Water Quality Index (IRWQIsc) and Geographic Information System (GIS). Water quality parameters such as electrical conductivity, pH, dissolved oxygen percentage, total hardness, turbidity, chemical oxygen demand, biochemical oxygen demand, nitrates, phosphates, ammonia, and fecal coliform were measured in winter and summer of 2013. The results showed that water quality indices at various stations were classified as poor and moderate, with a decreasing trend in water quality from upstream to downstream. The use of IRWQIsc and GIS facilitated better monitoring and management of river water pollution sources. Rezamohammadi et al.^4^ evaluated Sardabroud River’s water quality using IRWQIsc and biological indices. They found better quality in the cold season and declining conditions downstream due to municipal and agricultural pollution, emphasizing the need for a protection plan to preserve the river. Roshani-Sefidkouhi et al.^32^ assessed the water quality of the Talar River in Mazandaran in 2023 using the IRWQIsc and NSFWQI indices. Samples were collected from 10 points, and 11 physicochemical parameters were analyzed. The results showed that nitrate (NO_3_^−^) was within WHO standards, but phosphate (PO_4_^3^^−^) and COD occasionally exceeded permissible limits. Turbidity and EC consistently exceeded WHO and EPA guidelines. Both indices rated water quality as poor at all points, with some improvement in the summer (IRWQIsc) and winter (NSFWQI). The study concluded that agricultural and industrial pollution were the main causes of the declining water quality. Previous studies widely use water quality indices to assess surface water quality, yet further studies needed to understand the pollution dynamics along sequential monitoring stations. Although many studies have applied indices such as IRWQI and WQI to assess surface water quality, most have focused on single points or broad regions, often overlooking gradual changes along the river continuum. Limited research has investigated pollution dynamics across sequential monitoring stations or assessed the cumulative buildup of pollutants along river courses. This study addresses these gaps by analyzing spatial variations in water quality along the Little Zab River using the IRWQIsc index. By identifying pollution hotspots and longitudinal trends, it presents a novel approach to understanding progressive pollution patterns, an aspect often overlooked in existing national and regional research.

### Scope and objective

The Little Zab River in northwestern Iran is a vital water source for agriculture, domestic use, and the Sardasht Dam. Monitoring its water quality is essential to assess human impacts and inform management strategies. This study utilizes the Iranian Water Quality Index (IRWQI) to evaluate spatial and temporal variations, identifying critical areas needing attention. One of the comprehensive water quality indices which is commonly used in Iranian rivers is the Iran Surface Water Resources Quality Index for Conventional Pollutants, which allows for a more precise examination of the impact of each parameter influencing water quality^33^. By assigning relative weights to each variable, this index enhances the ability to detect changes in water quality over time and space, making it useful for decision-making in management^34^. It was introduced to address the natural conditions and challenges of water sources in Iran^35^. The approach used in the development of IRWQI is based on combining key variables such as nitrate (NO_3_^−^), ammonium (NH_4_^+^), phosphate (PO_4_^3^^−^), biochemical oxygen demand (BOD), chemical oxygen demand (COD), total hardness (TH), fecal coliform (FC), pH, dissolved oxygen (DO), electrical conductivity (EC), and other relevant parameters^36,37^. This method, by considering the varying impacts of each of these parameters, provides a comprehensive view of water quality and is useful for analyzing trends in water quality variations in river systems, dam reservoirs, lakes, and groundwater sources^32^.

While international water quality indices are commonly used, this study applies the IRWQI, an index tailored to Iran’s specific surface water conditions. Developed with consideration of local climate, pollution types, and natural resources, IRWQI assigns weights to parameters most relevant to Iranian rivers^36,38^. It allows for more accurate assessment of local environmental impacts, including in the Little Zab River. Nevertheless, the results of this study can also be valuable to an international audience as the study offers a practical example of how localized indices can complement global ones, guiding water quality assessments in other regions with similar environmental contexts. By examining long-term water quality variations across successive stations, this study offers a broader approach than earlier works focused on single points or brief periods. Identifying spatial–temporal trends and point-source pollution are key features that set this research apart. The main objective of this study is to provide a detailed evaluation of spatial and seasonal variations in water quality across sequential monitoring stations along the Little Zab River using the IRWQI index. This research seeks not only to evaluate the present state of water quality but also to pinpoint specific sources of pollution and examine their possible effects on the ecological condition of the river. The practical significance of this work lies in its ability to support local authorities in prioritizing pollution control efforts, improving water quality management strategies, and informing policy development for sustainable river basin management. The novelty of this research stems from its sequential, station-by-station assessment over a long-term period (2015–2024), which allows for tracking the cumulative effects of pollution and self-purification processes along the river continuum, an approach that is less explored in previous regional studies.

## Material and methods

### Description of the study area

**Fig. 1:**
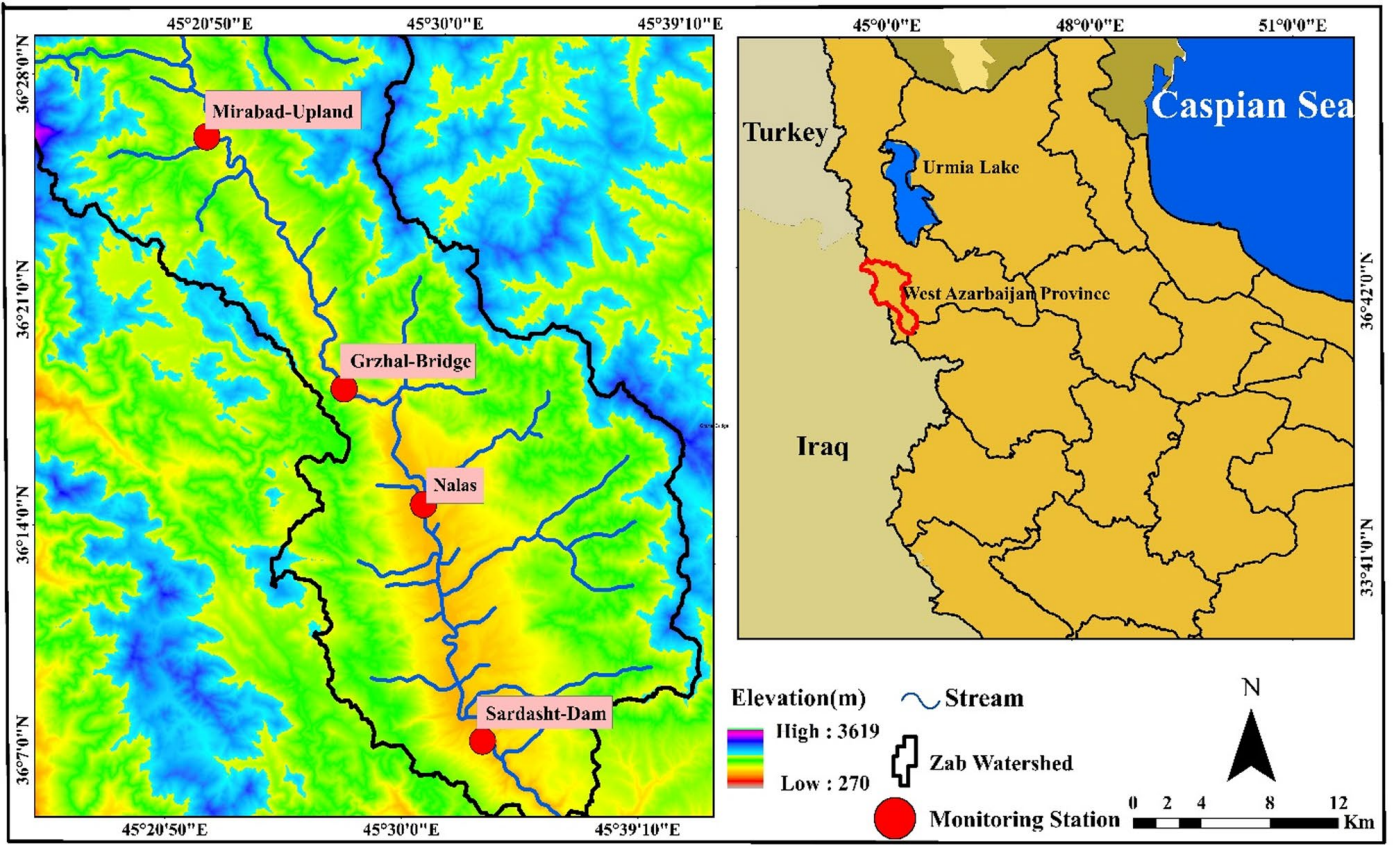
Geographical location of the study area and monitoring stations (Map processing and creation were carried out by the researchers using ArcMap within ArcGIS version 10.1^42^).

The Zab River originates from the northeastern highlands of the Zagros Mountain range and the Little Zab basin in Iran. After passing through Iran and Iraq, it flows for 302 km before joining the Tigris River in the Fatheh area (south of Mosul). The geographical range of this basin is between latitudes 35.16–36.79° North and longitudes 43.39–46.26° East. The Little Zab watershed covers an area of about 15,600 square kilometers, with 80% located in Iraq and 20% in Iran. The Little Zab watershed experiences a semi-arid to arid climate, characterized by hot, dry summers and humid winters. The average annual temperature varies, ranging from 22 °C in the south to 10 °C in the north. The average annual rainfall ranges from 350 mm in the south to 1500 mm in the north. According to studies, approximately 70% of the Little Zab watershed is covered by rangelands, with the remaining area used for agriculture. Additionally, Xerosols is the predominant soil type in these areas. Little Zab plays a significant role in the economy and environment of the region by supplying water for agriculture and livestock. Projects like the Kani Sev Dam are being implemented to transfer its water to Lake Urmia^39–41^ The flow pattern of the Great Zab and Little Zab rivers exhibits strong seasonal fluctuations, with peak flows occurring mainly due to snowmelt between April and May, and the lowest flow observed between July and December. The geographic location of the study area and monitoring stations is shown in Fig. 1.

### Research methodology

#### Water quality data

Four monitoring stations were selected based on the availability of continuous water quality data and their spatial distribution along the river course, ensuring representation from upstream to downstream sections. For the current research, water quality parameters, including physical, chemical, and biological data, were collected from the Environmental Protection Department of West Azerbaijan Province. These data included nitrate (NO_3_^−^), ammonium (NH_4_^+^), phosphate (PO_4_^3^^−^), biochemical oxygen demand (BOD), chemical oxygen demand (COD), total hardness (TH), fecal coliform (FC), pH, turbidity (Turb), and electrical conductivity (EC). The water quality data from 2015 to 2024 were collected from four consecutive monitoring stations (Mirabad-Upland, Grzhal-Bridge, Nalas, and Sardasht-Dam, in order from upstream to downstream) and were used in the analysis. Seasonal sampling with three replicates per station during cold and warm seasons was carried out by the Environmental Protection Department following standardized protocols for depth and timing to ensure data consistency and reliability. The dataset spans 2015–2024, with seasonal sampling and three replicates per station. The accuracy of data collection was supervised and verified by the Environmental Protection Department of West Azerbaijan Province. Prior to analysis, all datasets were subjected to quality control procedures including the identification and treatment of outliers and missing values to maintain data integrity.

Regarding the discharge value of the study river at the Grzhal hydrometric station, the minimum and maximum recorded values of average annual discharge were 16.10 and 86.71 cubic meters per second, respectively. The discharge range was calculated as 70.61 cubic meters per second. The mean discharge was 37.71 cubic meters per second, and the coefficient of variation was 42.93%, indicating relatively high flow variability at this station. The seasonal mean discharges were 14.75 in autumn, 31.91 in winter, 89.22 in spring, and 15.34 in summer, with the highest value observed in spring and the lowest in autumn. At the Brisoo hydrometric station, the measured discharge values ranged from 18.83 to 100.26 cubic meters per second, resulting in a range of 81.43 cubic meters per second. The mean discharge was 41.38 cubic meters per second, and the coefficient of variation was 45.84%, suggesting considerable variability in flow. The seasonal mean discharges were 16.18 in autumn, 38.25 in winter, 95.74 in spring, and 15.30 in summer, with the highest value occurring in spring and the lowest in summer.

The Table 1 presents the seasonal variations of four hydromorphological factors (depth, width, flow velocity, and slope) in the Zab River across different seasons of the year^43^.

**Table 1:** Average values of hydromorphological variables of the studied river across different seasons.

Factor	Spring	Summer	Autumn	Winter
Depth (cm)	69.05	49.74	42.00	62.50
Width (m)	57.38	20.53	20.00	28.42
Flow velocity (m/s)	0.85	0.66	0.50	1.19
Slope (%)	1.28	1.30	1.30	1.50

According to Table 1, the river depth is highest in spring (69.05 cm) and decreases to its lowest in autumn (42 cm), rising again in winter (62.5 cm). The river width also peaks in spring (57.38 m) and reaches its lowest in autumn (20 m), showing a sharp decline from spring to summer and autumn. Flow velocity is highest in winter (1.19 m/s) and lowest in autumn (0.50 m/s). The river slope shows less variation, but is highest in winter (1.50%) and lowest in spring (1.28%). Overall, spring is characterized by the greatest river depth and width, while in winter, despite narrower width compared to spring, flow velocity and slope are at their maximum. Autumn shows the lowest values for depth, width, and flow velocity, which may indicate reduced water levels and a decline in the river’s self-purification capacity during this season.

#### Iran’s surface water quality index (IRWQISC)

Various methods have been studied globally for assessing the quality of surface water, and among them, water quality indices are one of the most widely used and simple methods^16,44,45^. In the water quality index approach, a large amount of water quality data is integrated to a single index, which, based on the grading scale of each method, can indicate the classification of water quality status^46^. Some water quality indices, such as the National Sanitation Foundation, rely on a limited and specific set of data and parameters that apply universally. On the other hand, some indices, such as the Canadian Council of Ministers of the Environment water quality index, do not have this limitation and dependency^47,48^. This is a scientific communication tool that monitors multivariate water quality data in relation to a water quality reference set by the user, which the IRWQI_SC_ index belongs to the first group^49^. In the present study, changes in the values and the index of common water quality parameters (IRWQI_SC_) for surface water resources in the Little Zab River have been evaluated. Raw water quality parameters were converted to corresponding index scores using established ranking curves as defined by the Environmental Protection Agency of Iran. The IRWQISC composite index was calculated through a weighted geometric mean approach implemented via Microsoft Excel.

The common parameters used in the IRWQISC water quality index, approved by the Environmental Protection Agency of Iran, include an index of 11 water quality parameters, along with the unit of measurement for each, as shown in Table 2. The weights assigned to each parameter in calculating the IRWQISC were determined based on the standards established for surface waters in Iran^35^.

**Table 2:** Water quality parameters used in the calculation of the IRWQI index and their weights.

Water quality parameter	Unit	Weight
BOD_5_	mg/l	0.117
COD	mg/l	0.093
Dissolved Oxygen Saturation	% Saturation	0.097
Electrical conductivity	μS/cm	0.096
Fecal Coliform	No./100 ml	0.140
NH4 +	mg/l	0.090
NO3-	mg/l	0.108
PO43-	mg/l	0.087
Total Hardness	mgCaCO3/l	0.059
Turbidity	NTU	0.062
pH	Standard Unit	0.051

Initially, the values of the parameters used are specified, and then the weight of each parameter is considered in the calculations based on Table 2^50^. The index value for each parameter is then calculated using ranking curves within the numerical range from 0 to 100. Finally, the index is computed using Eqs. 1 and 2:

1 $$IRWQI_{{sc}} = \left[ {\prod _{{i = 1}}^{n} I_{i} ^{{W_{i} }} } \right]^{{\frac{1}{\gamma }}}$$

2 $$\gamma ={\sum }_{i=1}^{n}{W}_{i}$$

where n is the number of water quality parameters, Ii is the index value for the i-th parameter from the ranking curve, γ is the geometric weighted mean, and Wi is the weight of each water quality parameter. To describe water quality based on the index value, Table 3 is used^26^.

**Table 3:** Descriptive equivalent of the calculated water quality index.

Index value	Water condition
< 15	Very bad
15–29.9	Bad
30–44.9	Fairly bad
45–55	Moderate
55.1–70	Fairly good
70.1–85	Good
> 85	Very good

#### Statistical assessment

After calculating the IRWQI index values at various sampling locations, the Shapiro–Wilk normality test and Levene’s test were used to check for data normality and homogeneity of variance across the different sampling stations^51,52^. Normality Q-Q plots were created to visualize the overall distribution pattern of the data. Given the non-normal distribution and variance heterogeneity among water quality data, the Kruskal–Wallis non-parametric test was employed to assess significant differences in IRWQI values across sampling stations along the Little Zab River^53,54^. This approach is appropriate for comparing multiple independent groups without assuming normality. The Kruskal–Wallis test (non-parametric ANOVA) is used to compare the distributions of data across more than two independent groups. To show the differences in the IRWQI values between sampling stations, boxplots were used. Data analysis was carried out using the R programming language, and the results were provided in the form of output files and graphical plots^55,56^.

## Results and discussion

Summary statistics of water quality parameters from the four monitoring stations along the Zab River are presented in Table 4.

**Table 4:** Summary statistics of water quality parameters across four monitoring stations on the Zab River.

Station	Stat/WQ parameter	BOD	COD	DO	Cond	NO_3_^−^	PO4	Turb	pH
Mirabad-Upland	Mean	2.61	9.10	8.60	296.00	4.72	1.21	60.00	7.88
	Median	2.55	7.50	8.16	300.00	3.40	1.00	60.00	8.12
	SD	1.43	6.40	1.21	61.52	5.92	0.96	59.40	0.56
	Sample Var	2.04	40.94	1.47	3785.22	35.06	0.93	3528.00	0.32
	Kurt	0.81	2.79	2.01	0.77	7.53	− 0.35	NA	0.34
	Skew	0.99	1.48	1.50	0.71	2.54	0.82	NA	− 1.13
	Min	1.00	2.00	7.17	202.00	0.10	0.06	18.00	6.65
	Max	6.00	28.00	11.30	450.00	23.00	3.10	102.00	8.64
Grzhal-Bridge	Mean	2.55	10.50	8.34	304.62	4.51	0.99	8.00	7.78
	Median	2.00	8.50	7.88	314.00	3.30	0.50	8.00	7.91
	SD	1.76	9.26	1.32	54.58	5.29	1.43	NA	0.68
	Sample Var	3.10	85.79	1.74	2978.55	28.03	2.04	NA	0.46
	Kurt	0.72	10.64	2.33	1.83	8.10	10.41	NA	0.08
	Skew	0.94	2.97	1.60	0.84	2.56	3.07	NA	− 0.83
	Min	0.00	1.00	7.20	208.00	0.10	0.06	8.00	6.20
	Max	7.00	46.00	11.37	456.00	21.50	5.80	8.00	8.73
Nalas	Mean	2.81	20.33	8.10	315.63	4.89	0.83	565.00	7.82
	Median	2.30	9.50	8.00	317.00	2.90	0.45	565.00	7.74
	SD	2.29	38.68	1.88	97.31	8.90	0.88	NA	0.45
	Sample Var	5.22	1496.00	3.53	9469.50	79.25	0.77	NA	0.21
	Kurt	2.07	15.71	0.58	0.92	11.87	0.23	NA	0.37
	Skew	1.62	3.88	0.44	0.82	3.39	1.14	NA	− 0.56
	Min	0.30	1.00	5.49	158.70	0.10	0.06	565.00	6.89
	Max	8.00	171.00	11.30	549.00	34.00	2.60	565.00	8.56
Sardasht-Dam	Mean	3.03	14.02	8.40	286.18	4.59	0.56	96.00	7.91
	Median	3.00	12.00	7.97	289.00	2.25	0.44	42.00	8.02
	SD	1.75	9.81	1.45	73.47	7.87	0.32	113.12	0.52
	Sample Var	3.07	96.29	2.10	5397.25	61.90	0.10	127.96	0.27
	Kurt	0.36	0.90	2.85	0.83	9.31	− 1.31	NA	0.17
	Skew	0.63	1.07	1.61	0.42	3.08	0.64	1.66	− 0.65
	Min	0.00	1.00	6.20	129.00	0.10	0.20	20.00	6.74
	Max	8.00	40.00	12.29	489.00	33.00	1.00	226.00	8.90

An evaluation of the spatial variation in water quality parameters along the Zab River, ranging from the upstream station Mirabad-Upland to the downstream station Sardasht-Dam, reveals several notable trends. The mean BOD decreases slightly from 2.61 at Mirabad to 2.55 at Grzhal, then increases downstream to 2.81 at Nalas and 3.03 at Sardasht. COD follows a similar pattern, rising significantly from 9.10 at Mirabad to 20.33 at Nalas, then dropping to 14.02 at Sardasht. DO remains relatively stable across all stations (ranging between 8.10 and 8.60), with the lowest average observed at Nalas (8.10). Electrical conductivity (Cond) increases from 296.00 at Mirabad to a peak of 315.63 at Nalas, then declines to 286.18 at Sardasht. Nitrate (NO_3_^−^) levels fluctuate, reaching the highest average at Nalas (4.89) and slightly decreasing to 4.59 at Sardasht. In contrast, phosphate (PO_4_^3^^−^) shows a decreasing trend from the upstream to the downstream, with the highest mean at Mirabad (1.21) and the lowest at Sardasht (0.56). A sharp increase in turbidity is evident at Nalas, where the mean value reaches 565.00, compared to significantly lower values at other stations (e.g., 60.00 at Mirabad and only 8.00 at Grzhal). The pH remains nearly neutral across all stations, ranging between 7.74 and 8.12, with the highest mean at Mirabad (7.88) and the lowest at Nalas (7.82). As a conclusion, this comparison indicates that the Nalas station exhibits more critical water quality conditions, with markedly elevated COD, NO_3_^−^, and turbidity levels compared to the other stations.

### Statistical analysis of IRWQI values

**Fig. 2:**
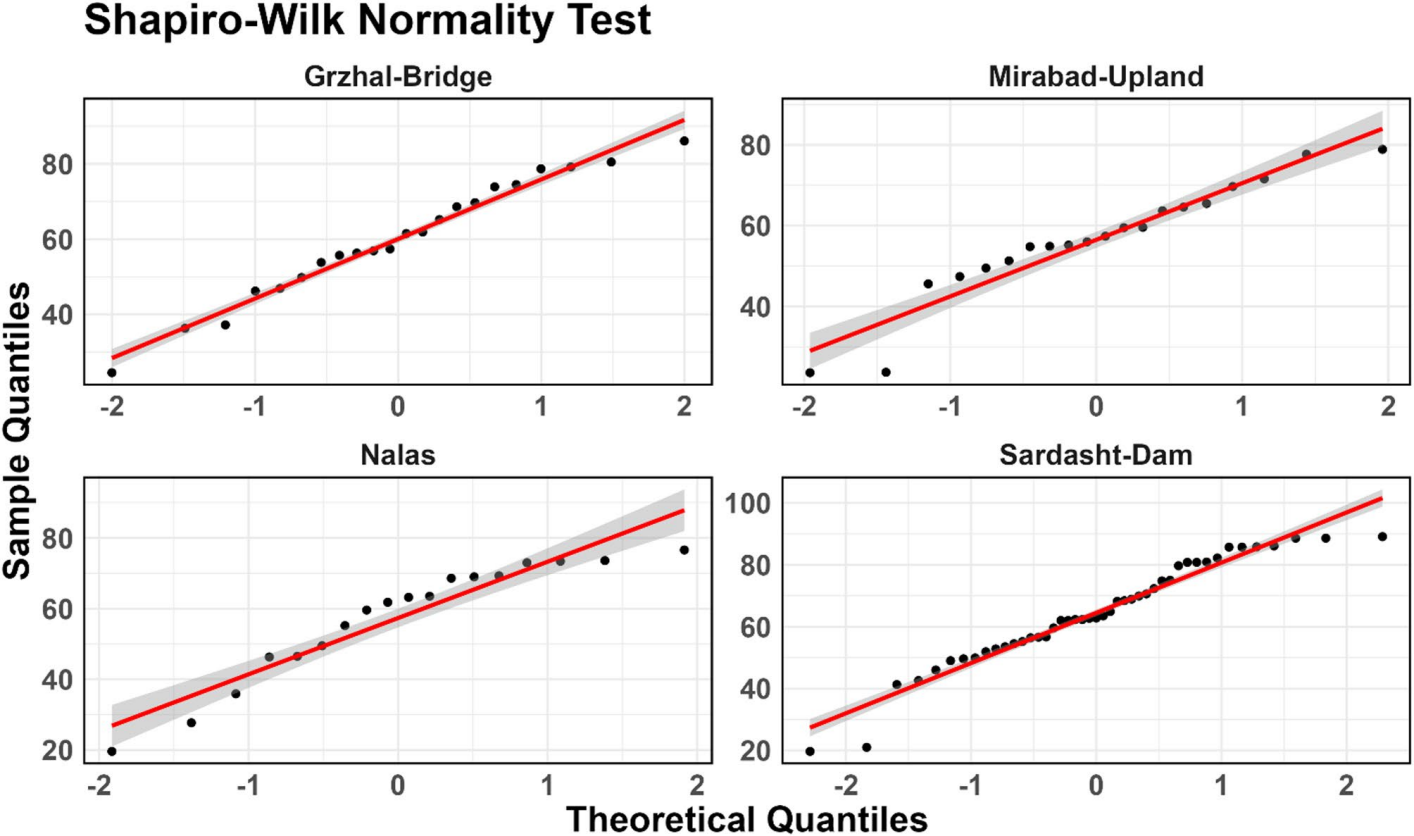
Graph of the results of the Shapiro–Wilk test regarding the normality of IRWQI values at different stations.

The Shapiro–Wilk test showed that the IRWQI values at the Grzhal-Bridge and Mirabad-Upland locations are normally distributed (*p*-value greater than 0.05) (Fig. 2). However, at the Nalas and Sardasht-Dam locations, the IRWQI values are not normally distributed.

The results of the Levene’s test showed that the test statistic is 0.3278, with a *p*-value of 0.8052. Since the *p*-value is greater than 0.05, it indicates the homogeneity of variances across the different sampling locations. The results of the Kruskal–Wallis test regarding the significance of changes in IRWQI values at sequential stations showed that the test statistic is 4.245, with degrees of freedom equal to 3 and a *p*-value of 0.236. Since the *p*-value is greater than 0.05, no significant difference was observed between the IRWQI values at the stations studied.

**Fig. 3:**
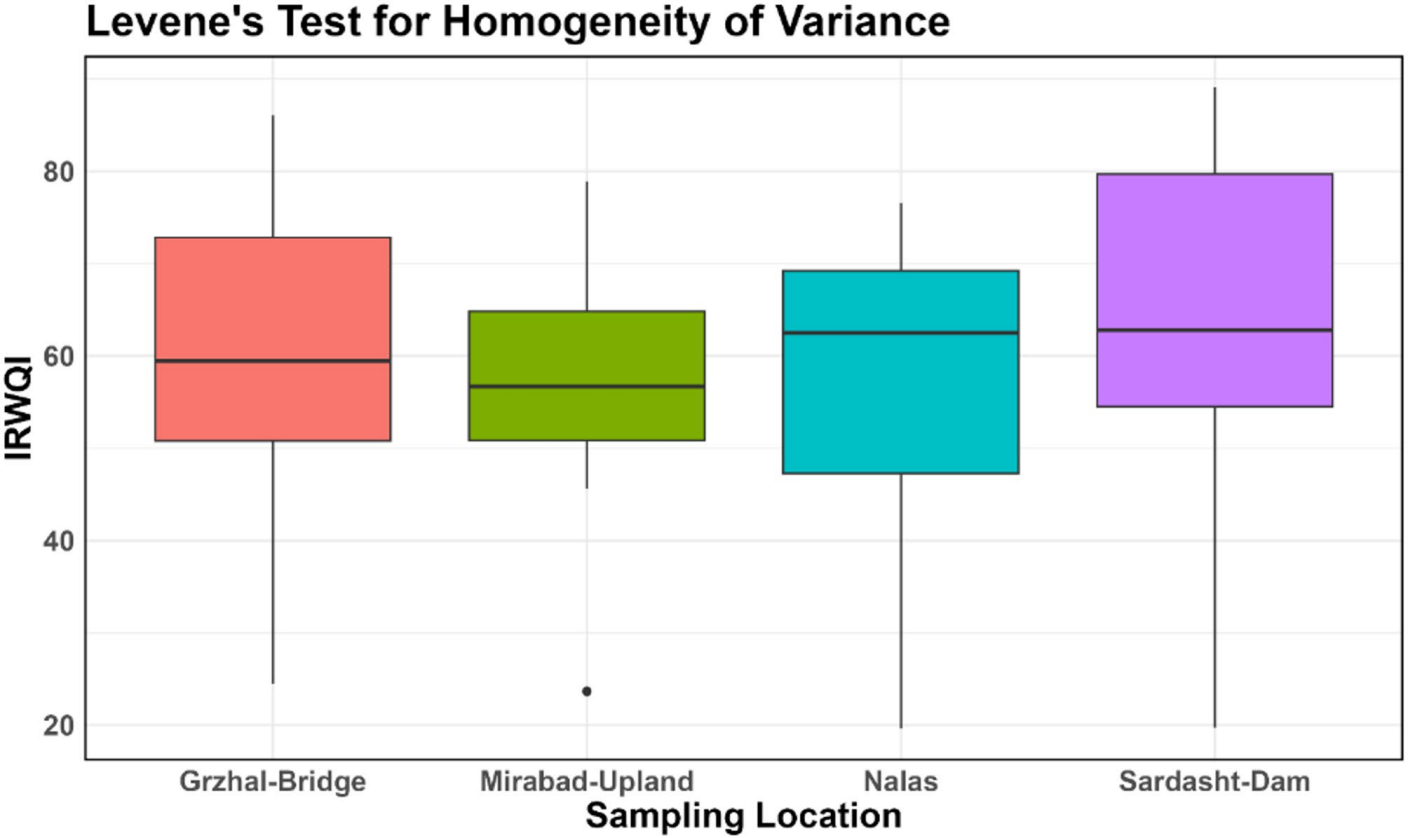
Box plot of changes in IRWQI values at different sampling stations.

The boxplot of IRWQI values at four monitoring stations along the study river has been shown in Fig. 3.

According to the Fig. 3, The IRWQI value at the Mirabad-Upland station, located upstream, is 56.51, indicating relatively poor water quality compared to the other stations. This is likely due to direct wastewater discharge from the Chaku village into the river.

At the Grzhal-Bridge station, the IRWQI value increases to 60.04. This increase could be due to natural self-purification processes, the input of lateral flows with better quality, or a reduction in the incoming pollution load in this section of the river, which improves water quality.

At the Nalas station, the IRWQI value decreases to 57.35. This reduction may indicate the entry of point or non-point pollution sources in this section of the river. Human activities such as wastewater discharge, agricultural runoff, or changes in lateral flows could be factors contributing to the decline in water quality. Additionally, a reduction in flow discharge can increase pollutant concentrations, further decreasing water quality. The presence of the Vavan village upstream of the Nalas station, with its high wastewater output, leads to noticeable changes in water color and odor at the point where wastewater enters the river, significantly affecting water quality.

The highest IRWQI value of 64.46 was observed at the Sardasht-Dam station. This increase may be due to the river’s self-purification effect, sedimentation of pollutants along the river’s course, or the inflow of higher-quality water into this area.

### Changes of IRWQI over monitoring stations

**Fig. 4:**
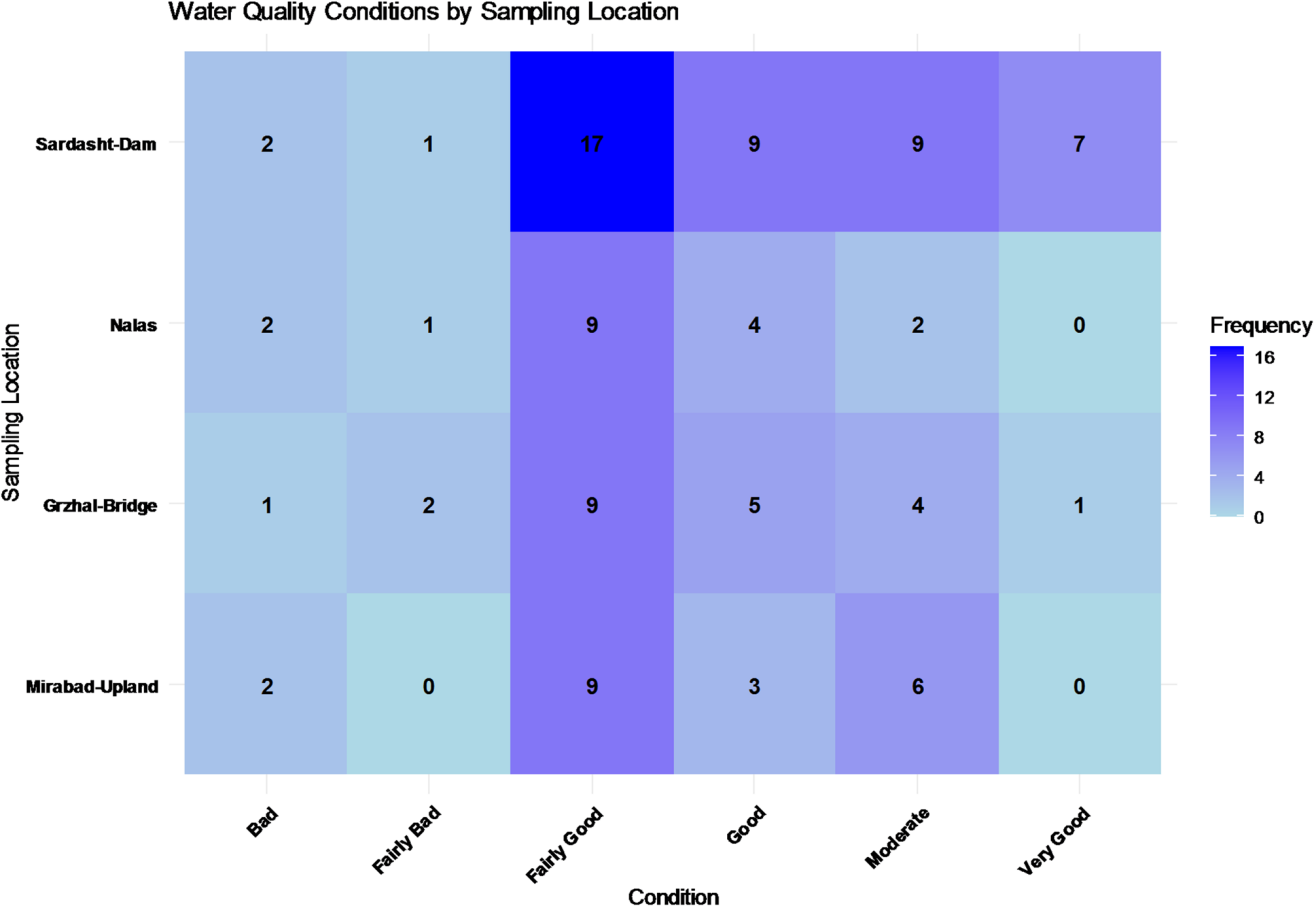
Comparison of water quality status frequency based on IRWQI index at different stations.

The comparison of water quality status frequency based on the IRWQI index at different stations is presented in Fig. 4.

Based on the analysis of water quality status at various monitoring stations from upstream to downstream (Fig. 4), it is observed that at the Mirabad-Upland station, the first station upstream, the water quality is mostly reported as “Fairly Good” and “Moderate.” At the Grzhal-Bridge station, located downstream of Mirabad-Upland, the water quality status is mostly within the “Fairly Good” and “Moderate” range, with a few instances of “Good” and even “Very Good.” This indicates that the water quality at this station is generally better than at the upstream station, but some quality issues like “Bad” and “Fairly Bad” are still observed.

At the Nalas station, the water quality status is mostly towards “Bad” and “Fairly Bad.” This indicates a decline in water quality from upstream stations to this station. In fact, the Nalas station, being at the lowest point, is likely affected by pollution from human activities or more severe environmental changes, which have led to the degradation of water quality. At the Sardasht-Dam station, located at the lowest point among the stations, water quality is mostly reported as “Fairly Good” and “Moderate,” with some instances of “Good“ and “Very Good.” This indicates that in the downstream stations, improvements in water quality are observed, but occasional occurrences of “Bad” and “Fairly Bad” suggest that specific factors may be influencing water quality variations.

Overall, it can be concluded that from upstream to downstream, water quality has significantly decreased at some stations, which could reflect the impact of pollution, hydrological changes, or human activities in the lower reaches.

Regarding the results shown in Fig. 4 it should be noted that in high-flow months (December–May), the Mirabad-Upland and Grzhal-Bridge stations had 8 samples each, Nalas had 7 samples, and Sardasht-Dam had 18 samples. In low-flow months (June–November), the Mirabad-Upland and Grzhal-Bridge stations had 10 and 11 samples respectively, Nalas had 9 samples, and Sardasht-Dam again had 18 samples. Sampling in the Mirabad-Upland, Grzhal-Bridge, and Nalas stations was almost equal in both high-flow and low-flow months, but Sardasht-Dam had more samples taken in the high-flow months.

In the Sardasht-Dam station, more samples were collected during high-flow months, while other stations had a balanced distribution of sampling across both high-flow and low-flow months. Overall, the frequency of water quality assessments at the stations may be related to the frequency of sampling. Therefore, assessing the trends in water quality variations (without considering frequency) can provide a better understanding of the water quality variations along the river (from upstream to downstream, including Mirabad-Upland, Grzhal-Bridge, Nalas, and Sardasht-Dam).

Khalife & Khoshnazar^24^ noted that no station in the Zarrineh River’s catchment area classified as the “Very Bad” or “Very Good” categories, with spring showing only marginally poor water quality at one station. In contrast, our study identified a more diverse range of water quality statuses, from “Fairly Good” to “Bad,” indicating significant variations in water quality across stations, particularly influenced by human activities such as wastewater discharge. This contribution shows the regional specificity of water quality variations and their underlying causes, such as direct contamination from villages like Vavan which affect the water quality at Nalas monitoring station. Shahsavar et al.^26^ found that the IRWQI index for the Karde Dam showed “Fairly Good” or “Moderate” water quality in different seasons. In contrast, this study reports a broader range of water quality from upstream to downstream, revealing an observable decline at the Nalas station, where human activities are likely contributing to poor water quality, which shows the regional variation in the effectiveness of natural self-purification processes, which might be more pronounced in some areas, as seen at Sardasht-Dam (downstream monitoring station).

Unlike the narrower IRWQI range in the Zarrineh River^24^, our study shows greater variability, from “Fairly Good” to “Bad”, with severe degradation at Nalas, likely due to local pollution sources like Vavan village. Seifollahi et al.^31^ and Roshani-Sefidkouhi et al.^32^ noted downstream water quality decline, which aligns with our Nalas results, but the recovery at Sardasht-Dam in our study suggests possible dilution or hydrological improvement not highlighted in their results. While Roshani-Sefidkouhi et al.^32^ reported consistently poor quality in the Talar River, our results show recovery at Sardasht-Dam, pointing to differing pollution sources, self-purification capacity, or reservoir impacts between regions.

The results of this study indicate that water quality along the river course shows significant fluctuations, influenced by the direct discharge of domestic wastewater, agricultural runoff, and natural variations in water quality due to self-purification processes. These findings are consistent with studies such as Roshani-Sefidkouhi et al.^32^ and Rezamohammadi et al.^4^ both of which attribute downstream water quality degradation to urban and agricultural pollution. However, unlike previous studies that have assessed water quality at a single point or based on annual averages, this research examines the gradual changes in water quality along the river, which can be highly useful in identifying critical zones and formulating local management policies. A comparison of the results of this study with recent research such as Gabr & Soussa^30^ in the Nile Delta and Kwon & Jo^29^ in South Korea reveals that the pattern of declining water quality due to human activities is a common phenomenon in many rivers under urban and agricultural pressure. However, the present study focuses on the gradual trend of water quality variations and its spatial analysis across consecutive monitoring stations, which can be valuable for designing more efficient and targeted monitoring networks.

### Changes of IRWQI over months and seasons

Based on the hydrological conditions of the study area, in the wet months (January, February, March, December), which fall in the spring and winter seasons, water quality is mostly in the “Good” or “Fairly Good” status. For example, in stations such as ”Mirabad-Upland” and “Sardasht-Dam,” water quality is often reported as “Good‚ or “Fairly Good” during these months. This indicates that in the wet seasons, due to higher water flow and possibly fewer pollutants compared to dry seasons, water quality tends to improve, meaning that during rainy seasons, water quality improves. In contrast, in the dry months (June, July, August, September, October), which belong to the summer and fall seasons, water quality is usually reported as “Bad” or “Fairly Bad.” For instance, in stations such as “Mirabad–Upland” and “Grzhal–Bridge,” water quality is more often observed in the “Bad” or “Fairly Bad” status during these months. This may indicate an increase in pollutants and a reduction in water flow, which leads to a decline in water quality during these months. During these periods, water scarcity and increased evaporation could lead to a higher concentration of pollutants in water sources. Similar to Shourian et al.^22^ who emphasized water quality improvement with fall water releases, our results show better water quality in wet months likely due to increased flow and dilution effects that reduce pollutant concentrations. This seasonal variation indicates the importance of hydrological conditions in regulating water quality dynamics. The observed decline in water quality during dry months aligns with Rezamohammadi et al.^4^ who reported lower water quality downstream linked to pollution accumulation and reduced flow. Our results reinforce how decreased discharge and pollutant concentration intensification during dry seasons worsen water quality. Unlike the consistently poor water quality reported by Roshani-Sefidkouhi et al.^32^ regardless of season, our study shows seasonal recovery in some stations, suggesting that local factors such as flow regimes and pollutant sources may lead to more variable temporal water quality patterns.

Overall, the data analysis shows that water quality is significantly related to the months and seasons. In wet seasons, water quality is generally better, while in dry seasons, especially in summer and fall, water quality decreases. For a more accurate interpretation, other factors such as pollutant levels, climatic conditions, and agricultural practices should also be considered, as these factors can have a significant impact on water quality throughout the year. The results show a distinct seasonal fluctuation, with water quality improving in the wet season. The impact of hydrological conditions on water quality, particularly the difference between high-flow and low-flow months, indicated the need for context-specific studies. However, this study introduces the different perspective of seasonal and hydrological influences, showing that water quality tends to improve during wet months, particularly at higher-flow month/seasons.

This study shows that river water quality is affected by domestic wastewater, agricultural runoff, and natural self-purification, causing significant fluctuations. Unlike previous studies focused on single points or annual averages, our sequential station analysis reveals gradual changes along the river, helping identify critical zones for management. Seasonal data indicate that high flows dilute pollutants and improve quality, while low flows concentrate pollutants and worsen conditions, requiring targeted actions. Significant differences, especially downstream, emphasize the need for location-specific policies to tackle pollution effectively.

### Implications and management recommendations

The results of this study can serve as a foundation for designing management strategies aimed at improving water quality at critical points along the river. For example, identifying the Nalas station as a location with a significant decline in water quality could justify the implementation of continuous monitoring programs, rural wastewater management, and agricultural runoff control upstream of this station. Additionally, the observed increase in the IRWQI index at the Sardasht-Dam station is likely due to natural self-purification processes and pollutant sedimentation, which should be further investigated through complementary studies such as sediment analysis and seasonal water quality assessments. Overall, the results revealed the need for a comprehensive, location-specific approach to managing water quality in mountainous rivers.

### Limitations and future research directions

Although the IRWQI integrates various water quality parameters, it does not identify specific pollutant sources or chemical compositions. The analysis captures seasonal variations but overlooks short-term pollution events. Land-use changes, unregulated wastewater, and climate variability were not modeled, limiting generalization. Future research should use advanced tracing techniques and high-resolution modeling to pinpoint pollution sources and assess climate change impacts on water quality. Additionally, evaluating policy effectiveness can support better pollution control strategies in the region. Given the data-driven nature of the IRWQI index and the limitations related to continuous real-time data in this study, it is recommended that future research employ machine learning models to predict water quality index values under various conditions. This approach can complement field-based analyses and also enable the simulation of different management scenarios or land use changes.

A key limitation of this study is the lack of sensitivity analysis due to insufficient modeling data and resources. Future research should consider using integrated models like Qual2K or SWAT with optimization algorithms to better evaluate indicator performance and system response.

## Conclusion

The Little Zab River is vital for agriculture, domestic use, and the Sardasht Dam in northwestern Iran, making water quality monitoring crucial. This study uses the Iranian Water Quality Index (IRWQI) to assess spatial and temporal variations at monitoring stations along the river, aiming to evaluate water quality variations from upstream to downstream. The statistical analysis of IRWQI values showed a different pattern of water quality across various stations. The Kruskal–Wallis test results indicated no significant differences in IRWQI values among the different water quality monitoring stations. Box plot analysis revealed that water quality at the Mirabad-Upland station was relatively poor due to wastewater discharge, while the Grzhal-Bridge station showed some improvement in water quality, likely related to natural self-purification processes. Changes in water quality across the stations were noteworthy. At the Mirabad-Upland station, water quality ranged from “Fairly Good” to “Moderate.” Downstream, at the Grzhal-Bridge station, water quality improved slightly, but instances of “Bad” and “Fairly Bad” quality were still observed. The Nalas station exhibited a decline in water quality, predominantly falling under “Bad” and “Fairly Bad” categories, which was attributed to human activities and pollution. At the Sardasht-Dam station, water quality improved again, with most values falling under “Fairly Good” and “Moderate,” although “Bad” and “Fairly Bad” instances were also noted. Monthly and seasonal variations in water quality revealed that during the wet months (December–May), water quality was better, with Mirabad-Upland and Sardasht-Dam stations reporting “Good” or “Fairly Good” status. In contrast, during the dry months (June–November), a decline in quality was observed, particularly at Mirabad-Upland and Grzhal-Bridge stations, where “Bad” and “Fairly Bad” quality prevailed. These seasonal changes indicated fluctuations in pollutant levels and water flow, with improved water quality linked to increased flow in the wet seasons. Despite these differences, the trend of water quality variations became clearer when considering all stations, revealing a decrease in water quality from upstream to downstream. This suggested the influence of various factors, such as pollution, human activities, and hydrological changes, particularly downstream in the river.

Overall, the water quality of the Little Zab River exhibited a distinct seasonal pattern, with better quality during the wet months and a decline during the dry months. This trend was primarily influenced by changes in water flow and pollutant concentrations, underscoring the need for continuous monitoring to identify pollution sources and effectively manage water quality throughout the year. This study effectively identifies critical zones of water quality decline and partial recovery along the Little Zab River using the IRWQI index. Results indicate the role of local pollution, hydrology, and seasonal patterns in shaping water quality. The results help guide targeted pollution control and restoration efforts. Future work should include variables like land use, population density, flow modeling, and sediment data. Incorporating machine learning and biological indicators, along with more frequent sampling, can enhance assessment and support sustainable water resource management.

## Data Availability

All data generated or analyzed during this study are included in this published article.
